# On the Preservation of Leeches

**Published:** 1860-10

**Authors:** J. Mill Frodsham

**Affiliations:** Physician to the Farringdon Dispensary


*On the Preservation of Leeches.* By J. Mill Frodsham, M. D., Physician to the Farringdon Dispensary.

Hospitals and vendors have endeavored to derive advantage from leeches by making them serve several times, and especially by preserving them; yet I believe even now a leech is seldom employed twice, the mode adopted and recommended in all books for cleansing them after they have been used being generally fatal. When I was house surgeon to the County Infirmary, Carlisle, I made numerous experiments to see how often and how long leeches could be employed and preserved without death; for this purpose a small glass vessel was used, in the bottom of which was placed about three inches of peaty earth, and twelve leeches, and then half filled with water, the mouth being secured with a coarse rag. These leeches lived in health for a year with only one or two deaths, many of them being employed twice in one day; but after being used, instead of putting them in salt, or vinegar and water, as usually done, they were placed for a few seconds in the camphor mixture of the Pharmacopœia, and afterwards washed in cold water, avoiding too much contact with the hands. But the apparatus *par excellence* for preserving leeches, is the one introduced by M. Vayson, an eminent French breeder, and called by him the "Domestic Marsh." This consists simply of an earthenware vessel in the form of a truncated cone reversed, the lower extremity of which is pierced with a few holes sufficiently narrow not to allow the leech to pass through. This vessel is filled with turfy earth, the leeches are placed in it, and they soon instal themselves as well as they can; then the orifice is closed with a coarse cloth. If desired to be sent to a great distance, the earth is wetted in all its thickness, and the vessel packed in a basket or box. If desired to preserve them on the same spot, the lower end of the vessel is placed in a tub, the water of which rises to the height of about four inches, and no further care is required. By the process of infiltration the lower strata of the "marsh" are soon wet through, the upper strata remaining dry; between these two extremes the leeches choose the zone most appropriate for them. M. de Quatrefages, by whom the above was brought before the French Academy, has kept and bred leeches in this way for two years with no deaths. This would lead us to believe that leeches do not live on the blood of animals, but on the infusoria.

The object of this communication is to recall attention to the fact that "*leeches* need *never be thrown away*"; and that hospitals and vendors may be induced to adopt this simple contrivance of M. Vayson, and thus make further experiments on the keeping and breeding of these very useful little creatures; for, as I have shown, they can be kept healthy and employed many times consecutively, if properly treated.

UPPER BEDFORD PLACE, Russell Square, W. C.

*Druggists' Circular.*

